# aEEG Use in Pediatric Critical Care—An Online Survey

**DOI:** 10.3389/fped.2020.00003

**Published:** 2020-01-24

**Authors:** Nora Bruns, Ursula Felderhoff-Müser, Christian Dohna-Schwake, Joachim Woelfle, Hanna Müller

**Affiliations:** ^1^Department of Pediatrics I, Neonatology, Pediatric Intensive Care, Pediatric Neurology, University Hospital Essen, University Duisburg-Essen, Essen, Germany; ^2^Division of Neonatology and Pediatric Intensive Care, Department of Pediatrics, University Hospital Erlangen, University of Erlangen-Nürnberg, Erlangen, Germany

**Keywords:** amplitude-integrated EEG, pediatric critical care, neuromonitoring, aEEG, continuous EEG, survey

## Abstract

**Background:** Evidence supporting continuous EEG monitoring in pediatric intensive care is increasing, but continuous full-channel EEG is a scarce resource. Amplitude-integrated EEG (aEEG) monitors are broadly available in children's hospitals due to their use in neonatology and can easily be applied to older patients.

**Objective:** The aim of this survey was to evaluate the use of amplitude-integrated EEG in German and Swiss pediatric intensive care units (PICUs).

**Design:** An online survey was sent to German and Swiss PICUs that were identified via databases provided by the German Pediatric Association (DGKJ) and the Swiss Society of Intensive Care (SGI). The questionnaire contained 18 multiple choice questions including the PICU size and specialization, indications for aEEG use, perceived benefits from aEEG, and data storage.

**Main results:** Forty-three (26%) PICUs filled out the questionnaire. Two thirds of all interviewed PICUs use aEEG in non-neonates. Main indications were neurological complications or disease and altered mental state. Features assessed were mostly seizures and side differences, less frequently height of amplitude and background pattern. Interpretation of raw EEG also played an important role. All interviewees would appreciate the establishment of reference values for toddlers and children.

**Conclusions:** aEEG is used in a large proportion of the interviewed PICUs. The wide-spread use without validation of data generates the need for further evaluation of this technique and the establishment of reference values for non-neonates.

## Introduction

Evidence supporting the use of continuous electroencephalography (EEG) in pediatric critical care has increased considerably in recent years. It has proven beneficial particularly after cardiac arrest, in patients with altered mental state and for the detection and treatment of seizures and monitoring of anticonvulsive treatment efficiency (1–5). Of special relevance is the detection of non-convulsive epileptic state, because this condition is associated with an increased risk of in-hospital mortality (2, 6). It has been shown that time from pediatric intensive care unit (PICU) admission to continuous EEG is associated with mortality in non-convulsive epileptic state (6). As continuous video-electroencephalograpy with the full 10–20 system of electrodes represents the gold standard, but remains a scarce resource requiring an epileptologist for interpretation, amplitude-integrated EEG appears to be a promising alternative. It is available in a large proportion of neonatal intensive care units and is easy to apply (7). aEEG has become standard of care in neonates after birth asphyxia for estimation of severity and prediction of outcome. It is also a valuable method for neonates with brain lesions and severe infections (8–11) as well as for preterm infants for the prediction of outcome (12–15). Also in adult intensive care, interest in aEEG is increasing recently (16–21). Its great advantage is that it can be operated and evaluated by the PICU team, independent from an epileptologist. After a short-term training period, staff is able to detect seizure activity and epileptic state in aEEG (22, 23). A recently published evaluation of aEEG use in our PICU showed that the main benefit came from detection and management of seizures and epileptic state (5). We also found that side differences were not detected in all patients with unilateral intracranial lesions, but if present, a unilateral process must be suspected. Similarly, background pattern was not necessarily altered in all patients with pathologies in conventional EEG but altered aEEG background pattern indicated pathological EEG and increased mortality. Adverse evolution of the background pattern over time was also significantly associated with death.

An obstacle to the further use of aEEG in non-neonates might be overcome by generating reference values and recommendations on how to deal with findings in the recording. As we noticed an increased use of this technique in our PICU over the past years (5), we initiated a survey in German and Swiss PICUs about their use of aEEG. The aim of the investigation was to assess the status quo of availability, indications, and use of amplitude-integrated EEG in pediatric critical care.

## Methods

German hospitals treating pediatric critical care patients were identified from the homepage of the German Pediatric Association (DGKJ, Deutsche Gesellschaft für Kinder- und Jugendmedizin) and the information given on the hospitals' websites. Swiss PICUs were identified from the homepage of the Swiss Society of Intensive Care (SGI, Schweizerische Gesellschaft für Intensivmedizin).

We contacted a 156 German and 7 Swiss PICUs via email in April and in September 2019. The email contained a link to an online multiple choice questionnaire (in German) on www.surveymonkey.com about the use of amplitude-integrated EEG in pediatric critical care patients.

The following items were assessed in the questionnaire: Multiple answers possible: specialization of PICU, availability of cerebral imaging, aEEG indications for all ages including neonates, use of aEEG in patients with altered mental state, qualities assessed in pediatric aEEG, classifications used for pediatric aEEG, perceived benefits at nights and at weekends, advantages of aEEG compared to conventional EEG, site and duration of data storage. Only one answer possible: name of hospital and city, PICU with NICU in the same ward or separate, number of PICU beds, availability of conventional EEG, number of available aEEG devices per hospital and per PICU, need for reference values. Each question included the possibility to add a comment.

Answers were exported from www.surveymonkey.com as Microsoft Excel^®^ files including raw data and summarized data. Analyses were made using Microsoft Office Excel^®^ Version 16.30. Median values were calculated for number of PICU beds and number of available aEEG devices per hospital and per PICU. For the remaining questions, the number of answers was counted and is presented as numbers (*n*) and percentages (%).

## Results

We received replies from 43 of 163 PICUs (26%) between April and September 2019. Forty-two PICUs were located in Germany, and one PICU was Swiss. Twenty (47%) PICUs belonged to university hospitals. Fourteen (33%) of 43 hospitals had PICUs without neonatal beds, whereas 29 (67%) hospitals had combined neonatal and pediatric intensive care units. The number of pediatric (excluding neonatal) intensive care beds ranged from 2 to 20 and was 7 at median (answered by *n* = 42, 98%).

**Figure 1:**
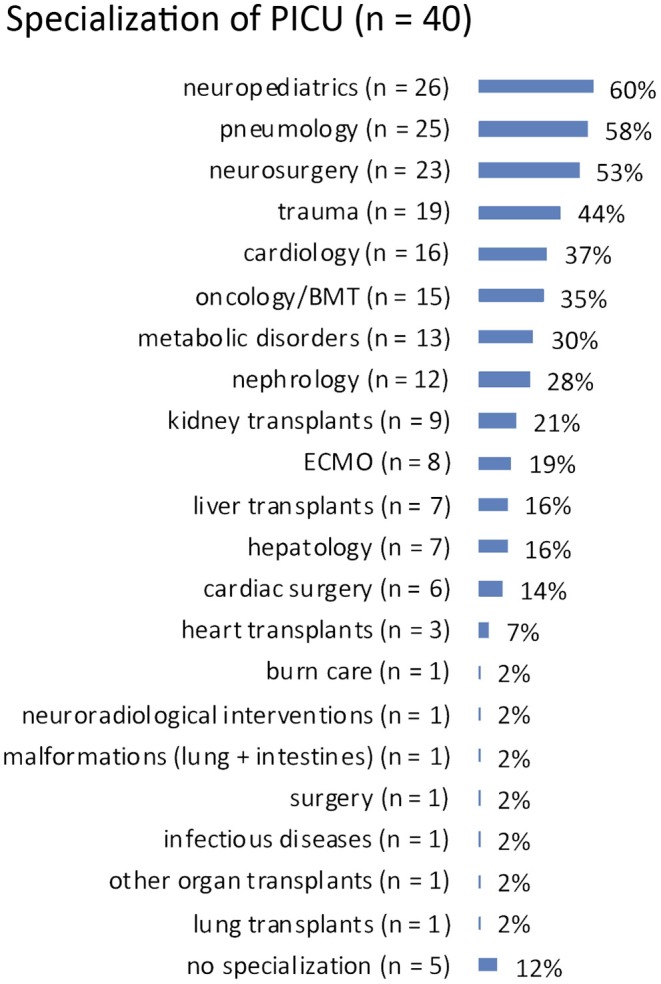
Specialization of interviewed PICUs.

Specializations: answered by *n* = 40 (93%). The responding PICUs reported heterogeneous specializations in neuropediatrics (*n* = 26, 60%), pneumology (*n* = 25, 58%), neurosurgery (*n* = 23, 53%), and trauma (*n* = 19, 44%), as most frequent specialties. See Figure 1 for more details.

### Availability of Cerebral Diagnostics and aEEG

**Figure 2:**
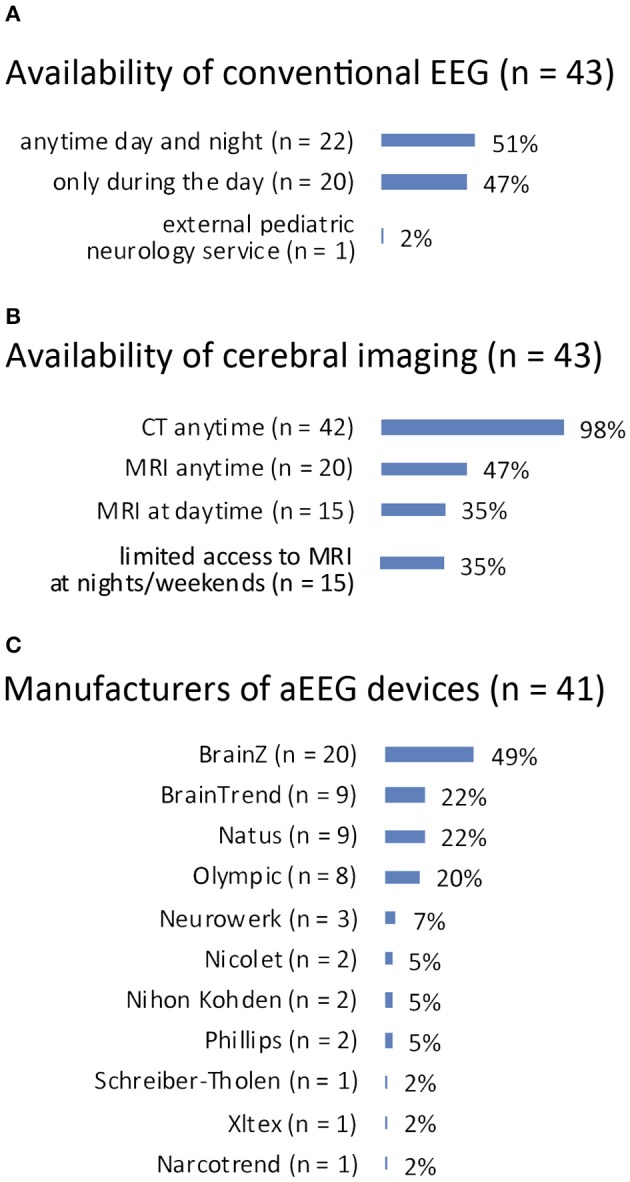
**(A)** Availability of conventional EEG. **(B)** Availability of cerebral imaging. **(C)** Manufacturers of aEEG devices.

Conventional EEG: answered by *n* = 43 (100%). Recording was available anytime during day and night in 22 (51%) and only at daytime in 20 (47%) PICUs. One (2%) PICU reported to make use of an external pediatric neurology service for conduction of EEG. See Figure 2A for details.

Cerebral imaging: answered by *n* = 43 (100%). Computed tomography for cerebral imaging was available in all 43 PICUs anytime, whereas magnetic resonance imaging (MRI) was available anytime in 20 (47%) PICUs and in 15 (35%) during daytime. Fifteen (35%) PICUs reported only limited access to MRI at night and at weekends. See Figure 2B for details.

Number of aEEG devices per children's hospital: answered by *n* = 43 (100%). Forty-two (98%) children's hospitals had aEEG devices available with a median number of two devices per hospital (range 1–12). One (2%) hospital did not have an aEEG monitor.

Number of aEEG devices per PICU: answered by *n* = 43 (100%). Forty (93%) PICUs reported they had access to aEEG devices with a median number of two devices per PICU (range 1–11). Three (7%) PICUs did not have access to an aEEG device.

Manufacturers: answered by *n* = 41 (95%). See Figure 2C for details.

### aEEG Indications and Use

**Figure 3:**
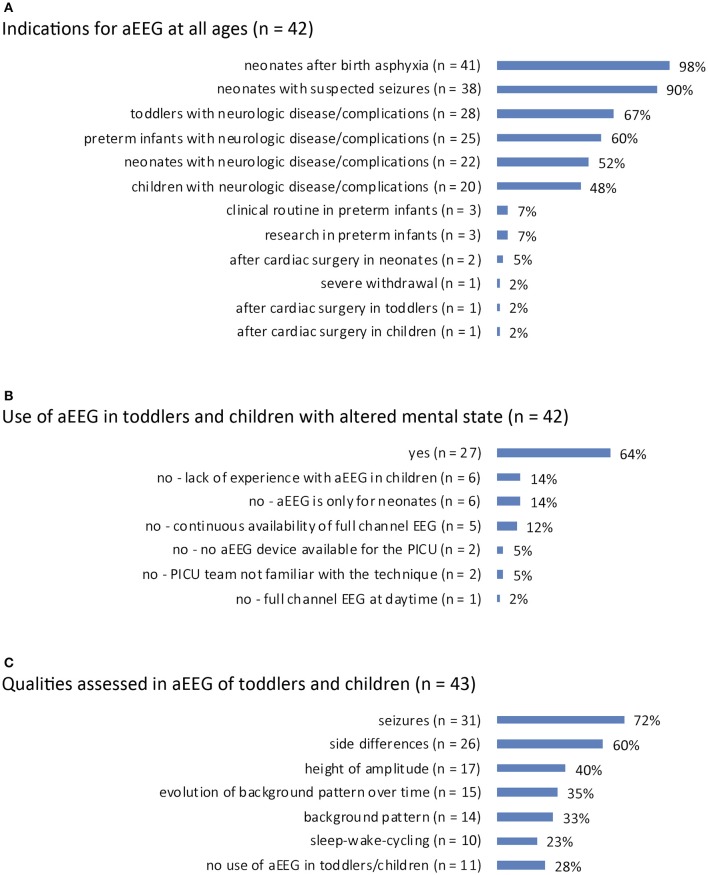
**(A)** Indications for aEEG recording for all age groups from preterm to child. **(B)** Use of aEEG in PICU patients with unexplained altered mental state. **(C)** Qualities assessed in aEEG when applied in toddlers/children.

Indications for aEEG: answered by *n* = 42 (98%). The most prevalent indications for aEEG use were conditions in neonates with birth asphyxia (*n* = 41, 98%) and neonatal seizures (*n* = 38, 90%). Neurologic disease or complications in preterm infants (*n* = 25, 60%), neonates (*n* = 22, 52%), toddlers (*n* = 28, 67%), or children (*n* = 20, 48%) were reported as further aEEG indications. Clinical routine or research purposes in preterm infants were rare indications (*n* = 3, 7% and *n* = 3, 7%). Two (5%) PICUs reported using aEEG after cardiac surgery in neonates, and one PICU (2%) used aEEG after cardiac surgery in toddlers and children, respectively. One (2%) PICU reported aEEG use for monitoring of severe withdrawal syndrome (Figure 3A).

Use of aEEG in toddlers/children with altered mental state: answered by *n* = 42 (98%). Twenty-seven PICUs (64%) applied aEEG in cases of unexplained altered mental state in toddlers or children. The reasons for not using aEEG in such cases were: lack of experience with aEEG in children (*n* = 6, 14%), a stance that aEEG is suitable only in neonates (*n* = 6, 14%), continuous availability of full channel EEG (*n* = 5, 12%), no device available for PICU patients (*n* = 2, 5%), the PICU team is not familiar with the technique (*n* = 2, 5%), and exclusive use of full channel EEG during daytime (*n* = 1, 2%) (Figure 3B).

### aEEG Interpretation in Toddlers and Children

Qualities assessed in toddlers'/children's aEEGs: answered by *n* = 43 (100%). Assessed qualities were seizures (*n* = 31, 72%), side differences (differences in hemispheric activity; *n* = 26, 60%), height of amplitude (*n* = 17, 40%), evolution of background pattern over time (*n* = 15, 35%), background pattern (*n* = 14, 33%), and sleep wake cycling (*n* = 10, 23%). Eleven (28%) PICUs reported they do not use aEEG in this age group (Figure 3C).

Classifications for interpretation: answered by *n* = 38 (88%). The classifications considered for interpretation were the assessment of the raw EEG curve (*n* = 26, 68%) and the classification by Hellström-Westas (*n* = 26, 68%). Two PICUs (5%) considered the Burdjalov classification for interpretation, and one (2%) PICU had their aEEGs assessed by a pediatric neurologist every 24 h.

Need for reference values: answered by *n* = 41 (95%). All answering PICUs considered reference values for toddlers and children helpful for interpretation.

### Attitudes Toward aEEG in Toddlers and Children

**Figure 4:**
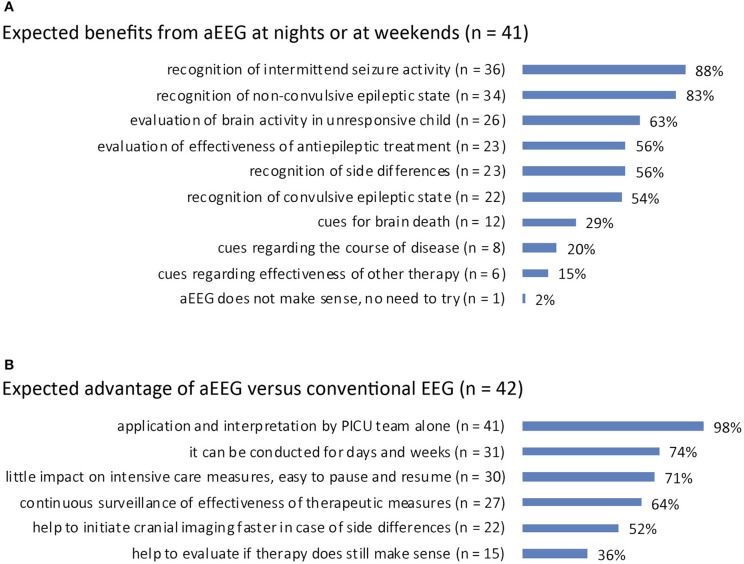
**(A)** Perceived benefit of aEEG over conventional EEG recording in pediatric ICU patients. **(B)** Expected benefit from aEEG in pediatric ICU patients at night/at weekends.

Expected benefits from aEEG at night or at weekends: answered by *n* = 41 (95%). Expected benefits were the recognition of intermittent seizure activity (*n* = 36, 88%), recognition of non-convulsive epileptic state (*n* = 34, 83%), and evaluation of brain activity in an unresponsive child (*n* = 26, 63%). Twenty-three (56%) interviewees expected aEEG to be useful for the detection of side differences and the evaluation of antiepileptic treatment, respectively, and for the recognition of convulsive epileptic state (*n* = 22, 54%). Less frequently expected benefits were cues for brain death (*n* = 12, 29%), cues regarding the course of disease (*n* = 8, 20%), and cues regarding the effectiveness of therapy (e.g., organ replacement therapy or vasopressor treatment; *n* = 6, 15%). One (2%) regarded aEEG as useless and thus did not attempt to try this method (Figure 4A).

Advantage of aEEG vs. EEG: answered by *n* = 42 (98%). The application and interpretation by the PICU team itself was named to be the expected advantage of aEEG compared to conventional EEG (*n* = 41, 98%). Further advantages reported were long duration of recording (for days and weeks; *n* = 31, 74%), little impact on intensive care measures (easy to pause and resume; *n* = 30, 71%), and continuous surveillance of treatment efficacy (*n* = 27, 64%). Twenty-two (52%) interviewees replied aEEG could help to initiate cerebral imaging faster and 15 (36%) said that aEEG could help to evaluate if ongoing therapy still makes sense (Figure 4B).

### aEEG-Data Storage

**Figure 5:**
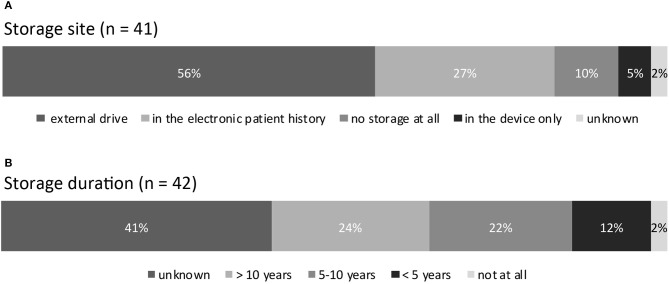
**(A)** Storage site of aEEG data. **(B)** Storage duration of aEEG data.

Storage site: answered by *n* = 41 (95%). See Figure 5A for details.

Storage duration: answered by *n* = 42 (98%). See Figure 5B for details.

## Discussion

We herewith report the results of an online survey investigating the use of amplitude-integrated EEG in Swiss and German pediatric intensive care units. To our surprise we found that, despite lack of validation, aEEG is presently being used for non-neonates by two thirds of the responding pediatric intensive care units. Beyond the established indications for aEEG in neonates—asphyxia and monitoring of suspected seizure activity—aEEG is used in cases of neurologic disease and complications for patients of all ages. Interviewees using aEEG reported the detection and management of seizures and the evaluation of brain activity in cases of altered mental state to be the main benefit of this method.

These results comply with results from an analysis of aEEG use in our PICU we recently published (5). We detected an increase in use of aEEG in our PICU in recent years with management of seizures being the main indication. Evidence supporting continuous EEG monitoring in children for the detection of non-convulsive seizures, after cardiac arrest, and with altered mental status is growing (1, 2, 6, 23, 24). Currently continuous full channel EEG is the standard technique for continuous cerebral function monitoring in pediatric critical care patients, but is not broadly available and requires specialist knowledge for interpretation (5). In our survey, just about half of all PICUs reported they could obtain a conventional (non-continuous) EEG anytime of the day. Given the limited access even to this routine technique, aEEG provides some advantages, that were also perceived by the participating PICUs: It is easily applied and interpreted by the PICU team and recordings can be continued for weeks if necessary. It can be paused and resumed, facilitating interventions. Additionally, staff in most children's hospitals is already familiar with aEEG devices from their neonatal intensive care units. Even though aEEG as a technique has its limitations such as reduced sensitivity for seizure detection and a potential for misdiagnosis due to artifacts, it seems to have its place where full-channel EEG is not available. Our data show that this is still the case in many PICUs.

As aEEG is in fact being used in pediatric intensive care patients, the question of how to interpret the tracing and how to deal with findings arises. Various classifications are available for preterm infants and neonates (25–28). However, no such classification exists for toddlers, children, and adults. This is also reflected by the fact that more than 10% of participants of the survey skipped the question about which classification they use for interpretation. A modified version of a neonatal classification (by Hellström-Westas) has been used in adults after cardiac arrest (18–21, 29). In accordance with these results, PICU staff almost equally often interpreted the raw EEG curve and used the classification by Hellström-Westas. The parameters most frequently assessed were seizures and side differences. These two parameters are independent from reference values and therefore can be interpreted quite safely, or after short-term training, respectively (22, 23). At the same time the presence of either of the two often leads to further diagnostic steps or therapy, making them important parameters. Less important to our interviewees were the height of amplitude, background pattern or evolution of background pattern over time. In our PICU cohort, we found that aEEG background was less sensitive to detect pathological changes compared to conventional EEG (5). This is likely caused by the fact that important EEG qualities like frequency and wave morphology are not displayed by aEEG. On the other hand, severe conditions were reflected by background pattern showing mainly flat trace or burst suppression pattern, which negatively correlated with survival. Similar findings were made in adults with acute brain injury: severely altered background pattern was associated with poor 6 month-outcome (17). Parietal aEEG bandwidth in teenagers and young adults with anti-NMDA receptor encephalitis was recently shown to be associated with outcome 12 months after admission to intensive care unit (16). These findings underline that aEEG background pattern reacts to pathological conditions. However, until further knowledge about physiological background in toddlers and children has been acquired, the present approach of most PICUs from our survey to focus on seizures and side differences seems reasonable.

**Figure 6:**
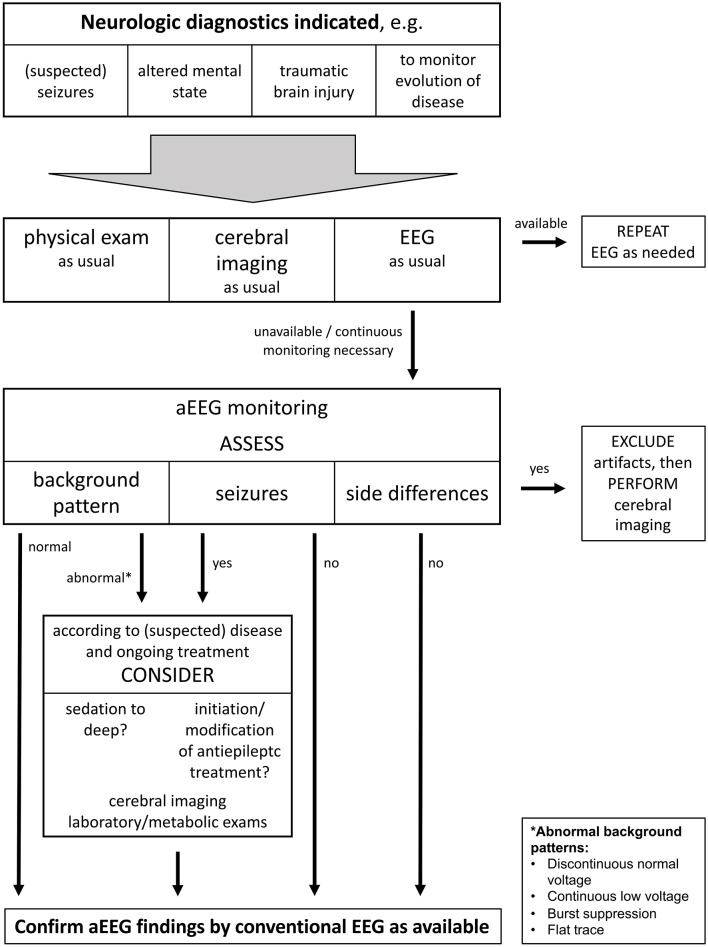
This flow chart shows a suggestion what an algorithm for clinical use of aEEG could look like. It starts with possible indications for cerebral function monitoring and suggests how to react to findings. It is important to note that aEEG is not meant to substitute but to complement standard diagnostic tools including conventional EEG. aEEG findings should be confirmed by conventional EEG according to availability.

The fact that aEEGs are being conducted in children generates the need to define how to react on findings, even before the establishment of reference values. Figure 6 displays a suggestion for clinical practice how the indication for pediatric aEEG could possibly be set and which actions ought to result from frequent findings. Continued research is required to show if reference values regarding height of amplitude and background pattern can be established for non-neonates, or whether interpretation of pediatric aEEG will remain limited to the detection of seizures, side differences, and severely altered electrocortical activity. Further, it needs to be determined if the conduction of aEEG provides benefits for non-neonatal patients regarding their courses of disease.

A limitation of this investigation is the low response rate. At the same time, university hospitals account for almost half of the participants of our survey, leading to an overrepresentation of these facilities. As a result of the organization of the German health care system, there are many hospitals with small children's departments and small pediatric intensive care units. Possibly, smaller hospitals do not use aEEG devices frequently or are not familiar with the technique and therefore did not feel addressed by the survey. Likely this bias leads to an overestimation of aEEG use. However, as larger hospitals tend to treat sicker children and apply new techniques earlier, chances are, that the trend to continuous cerebral function monitoring will spread to peripheral hospitals with some latency. We consider the findings of this survey as meaningful, because aEEG devices are already available in most children's hospitals with neonatal departments and aEEG is in fact already being used in critically ill pediatric patients. This highlights the need for further research to determine the prospects as well as the limitations provided by this technique.

To conclude, aEEG use still takes place mainly in neonatology, but will possibly find its place in pediatric critical care in the future. Pediatric aEEG interpretation remains challenging due to a lack of reference values and for now mainly focuses on seizures, side differences, and assessment of the raw EEG.

## Data Availability Statement

The raw data supporting the conclusions of this article will be made available by the authors, without undue reservation, to any qualified researcher.

## Author Contributions

NB and HM designed the questionnaire, conducted the survey, analyzed data, and designed figures. NB wrote the manuscript. HM, CD-S, UF-M, and JW helped to interpret data and prepare the manuscript. HM financed the online survey.

### Conflict of Interest

The authors declare that the research was conducted in the absence of any commercial or financial relationships that could be construed as a potential conflict of interest.
